# Robotic Services Acceptance in Smart Environments With Older Adults: User Satisfaction and Acceptability Study

**DOI:** 10.2196/jmir.9460

**Published:** 2018-09-21

**Authors:** Filippo Cavallo, Raffaele Esposito, Raffaele Limosani, Alessandro Manzi, Roberta Bevilacqua, Elisa Felici, Alessandro Di Nuovo, Angelo Cangelosi, Fabrizia Lattanzio, Paolo Dario

**Affiliations:** 1 Assistive Robotics Lab The BioRobotics Institute Scuola Superiore Sant'Anna Pontedera Italy; 2 Laboratorio di Bioinformatica, Bioingegenria e Domotica Istituto Nazionale di Riposo e Cura per Anziani Ancona Italy; 3 Sheffield Robotics Department of Computing Sheffield Hallam University Sheffield United Kingdom; 4 School of Computing, Electronics and Mathematics Plymouth University Plymouth United Kingdom

**Keywords:** social robotics, active and healthy aging, acceptability models

## Abstract

**Background:**

In Europe, the population of older people is increasing rapidly. Many older people prefer to remain in their homes but living alone could be a risk for their safety. In this context, robotics and other emerging technologies are increasingly proposed as potential solutions to this societal concern. However, one-third of all assistive technologies are abandoned within one year of use because the end users do not accept them.

**Objective:**

The aim of this study is to investigate the acceptance of the Robot-Era system, which provides robotic services to permit older people to remain in their homes.

**Methods:**

Six robotic services were tested by 35 older users. The experiments were conducted in three different environments: private home, condominium, and outdoor sites. The appearance questionnaire was developed to collect the users’ first impressions about the Robot-Era system, whereas the acceptance was evaluated through a questionnaire developed ad hoc for Robot-Era.

**Results:**

A total of 45 older users were recruited. The people were grouped in two samples of 35 participants, according to their availability. Participants had a positive impression of Robot-Era robots, as reflected by the mean score of 73.04 (SD 11.80) for DORO’s (domestic robot) appearance, 76.85 (SD 12.01) for CORO (condominium robot), and 75.93 (SD 11.67) for ORO (outdoor robot). Men gave ORO’s appearance an overall score higher than women (*P*=.02). Moreover, participants younger than 75 years understood more readily the functionalities of Robot-Era robots compared to older people (*P*=.007 for DORO, *P*=.001 for CORO, and *P*=.046 for ORO). For the ad hoc questionnaire, the mean overall score was higher than 80 out of 100 points for all Robot-Era services. Older persons with a high educational level gave Robot-Era services a higher score than those with a low level of education (shopping: *P*=.04; garbage: *P*=.047; reminding: *P*=.04; indoor walking support: *P*=.006; outdoor walking support: *P*=.03). A higher score was given by male older adults for shopping (*P*=.02), indoor walking support (*P*=.02), and outdoor walking support (*P*=.03).

**Conclusions:**

Based on the feedback given by the end users, the Robot-Era system has the potential to be developed as a socially acceptable and believable provider of robotic services to facilitate older people to live independently in their homes.

## Introduction

### Background

Longevity is one of the biggest achievements of modern societies and people aged 65 or older will account for 28.7% of the EU-28′s population by 2080, compared to 18.9% in 2015 [1]. Moreover, in 2011, 28.5% of Europe’s population older than 65 years of age were living their own homes, whereas for people older than age 85, the percentages were 49.5% for women and 27.8% for men [2]. Furthermore, 17.7% of Europe’s older citizens live in rural areas [2] where access to health care services can be limited. Older people generally prefer to remain in their homes [3], but they often are affected by multimorbidity [4], falls [5], loneliness [6], and the risk of malnutrition [7]. Considering these risk factors, the odds of institutionalization grows, thereby increasing the costs for health care services.

Considering all that, the World Health Organization and the Global Health Workforce Alliance are developing a strategy to plan effective human resources for health for the period 2016-2030. Although the health care labor market is growing, it is not clear if the number of health care workers will be able to meet the demand for older assistance [8]. In particular, in Europe by 2030, health assistance supply will fall short of demand to meet the health needs of an aging population [9].

In this context, robotics and other emerging technologies, such as ambient intelligence, are increasingly proposed as a potential solution to this societal concern [10]. In Europe, several research projects were founded under the ICT strand of the Seventh Framework Programme (FP7) [11] and EU Horizon 2020 Research and Innovation program [12], as discussed in [13].

Despite the growing interest in developing this type of technology for supporting older people, the target user must accept robots for them to be effective assistive technology tools for older people [14]. Unfortunately, one-third of all assistive technologies are abandoned within one year of use [15]. For this reason, the design and acceptability of service robots that interact with individuals and coexist in environments inhabited by humans are crucial aspects to overcome the resistance toward service robotics [16]. Furthermore, the concept of “trust” in the adoption of intelligent assistive technologies to assist aging in place by older adults is very important [17]. In this context, this paper shows the results achieved within the Robot-Era project, funded by the European Community’s FP7 (FP7/2007-2013), that aimed to investigate and demonstrate, among other things, the usability and acceptability by end users of a plurality of complete advanced robotic services, integrated into smart environments and experimented in realistic experiments.

### Related Works

The concept of robots that most people have is shaped by movies and science fiction, provoking a mismatch in what the robots of today can accomplish and what the movies portray [18]. For this reason, in recent years, many studies have been conducted to evaluate the acceptance of robots by older users [19-30]. In this section, the studies showing older adults’ feedback about robots are presented focusing on works comparable to the Robot-Era project.

Some of these studies were done involving older adults to explore their attitudes toward possible tasks that robots, in general, could perform in the home, but no robot was used in these studies [19,20].

Prakash et al [19] studied how human-likeness of the robot’s face influences the perceptions of robots by humans, involving 32 older adults. Data were collected using interviews and questionnaires; the outcomes showed a higher preference for the human-looking appearance of robots by older adults. However, no real robot was used in the study—participants’ imaginations were stimulated by pictures of robots such as Pearl nursebot, Nexi MDS, NAO, and Kobian.

Wu et al [20] involved 20 older persons with mild cognitive impairment to investigate their perceived attitude toward an assistive robot. The main outcome was that participants considered a robot useful to them in the future, but not in the present; they also deemed a robot to be useful for older people affected by frailty, loneliness, and disability. However, the limitation of this study was that older adults did not interact with a robot—their feedback was obtained by showing video clips and pictures of robots.

In other studies, a robot was presented to older people, but they did not have the opportunity to directly interact with it and their feedback was obtained after viewing a video clip or a live demonstration showing the potentialities of a robot [21-22].

Pino et al [21] presented the RobuLAB 10, a robotic mobile platform that provides seven robotic services for the cognitive and social support of older people. Ten older adults with mild cognitive impairment and eight healthy older adults were involved in the study to evaluate the acceptance of robots. The study employed a semistructured focus group and questionnaires. The results showed that participants positively perceived the potential benefits of the robot to support older adults at home, even if the intention to use was low. However, participants attended a live demonstration performed by a researcher and the robot was controlled remotely.

In a more recent study, on the basis of a demonstrative video of telepresence Kubi and Beam robots, Stuck et al [22] interviewed 14 older adults with mobility impairments who perceived the benefits of a robotics system for communication service. However, they mentioned some concerns about damage to themselves or the environment.

Other studies evaluated the acceptance of a service robot by older adults after they interacted with it in a controlled laboratory setting [23-25].

Fischinger et al [23] developed the Hobbit PT1 robot that could perform six tasks to support older adults. The acceptance was evaluated by 49 older users who interacted with the robot in a laboratory decorated as a living room. The outcome of the survey showed a positive reception by users. More than half of the sample could imagine having the robot at home for a longer period, although approximately half the participants were skeptical about its helpfulness. However, during the controlled laboratory user studies, the robot was not autonomous because a researcher remotely controlled it.

In another study, 33 older users interacted with a robot as a physical exercise coach that was appreciated as an exercise motivator by most participants [24]. Furthermore, a study with 16 healthy older adults was conducted in a controlled laboratory environment. The aim was to investigate their acceptance of robots for partner dance-based exercise. The results showed the robot was perceived as useful, easy to use, and enjoyable [25].

Cavallo et al [26] developed and tested an enhanced robotic platform, called ASTROMOBILE, which was integrated into an ambient intelligent infrastructure to provide a favorable independent living. Sixteen older users were involved. The robot was autonomous, and experiments were conducted in a domestic house. The ASTROMOBILE system provided three functional capabilities. The study was conducted as a focus group and live demonstration, but each participant tested at least one robotic capability. The results demonstrated a positive impression by older users and the utility of robotic services was appreciated.

Other studies focused on robot acceptance were conducted in actual environments [27-30]. Koceski et al [27] developed an assistive telepresence robot that was tested by 30 older adults in a nursing home. The results show that the functionalities provided by the telepresence robot system were accepted by potential users, but the robot was not autonomous because it was teleoperated by the user, both for navigation and for fetch and carry of a small object, and only three robotic services were provided. In addition, although the experiments were conducted in a real environment, it was a pilot study and the robotic system was not integrated into the daily routine of the nursing home.

Broadbent et al [28] investigated the effectiveness of the iRobi robot delivering telehealth care to increase adherence to medication and home rehabilitation, improve quality of life, and reduce hospital readmission compared with a standard care control group. A total of 25 older persons with chronic obstructive pulmonary disease used the robot, and the results showed that a homecare robot can improve adherence to medication and increase exercise, even if there were no significant differences in quality of life.

Finally, Orlandini et al [29] assessed the robustness and validity of the mobile robotic telepresence system Giraff as a means to support older persons and to foster their social interaction and participation. Cesta et al [30] evaluated the acceptance of the Giraff robot by two older persons in a long-term trial and received positive results. An overview of the related works is shown in Multimedia Appendix 1 (Overview of Related Works).

### Goal of This Study

As stated previously, the acceptance of robots by older users has been examined in many studies, but there are some limitations. First, in some studies, older individuals have expressed an opinion without interacting with a robot. Feedback was collected from users based only on pictures of robots [19,20], or a video clip showing the robot’s capabilities [22], or a live demonstration performed by a researcher [21]. Second, some studies involved a small number of participants [22], and those studies conducted with many older adults had some limitations because users attended a single live demonstration without direct interaction with a robot [21]. In some studies, the experiment was conducted with a “Wizard of Oz” methodology (experiment in which participants interact with a system that they believe to be autonomous, but which is controlled by a hidden person) [23], or the robot was teleoperated by the user [27]. Third, in some cases the robot was not autonomous [23,27] or was a stationary robot. Finally, in all considered studies, only one robot, working in a single environment, was used.

In this research, some of these limitations were overcome: (1) a total of 45 older adults extensively interacted directly with three robots to accomplish tasks, (2) three autonomous robots were used to cooperate between them in smart environments, (3) the experiments were conducted in three different environments (domestic, condominium, and outdoor areas), (4) six robotic services were provided by the Robot-Era system, and (5) each Robot-Era service was tested by 35 older users.

## Methods

### Robot-Era Architecture

**Figure 1:**
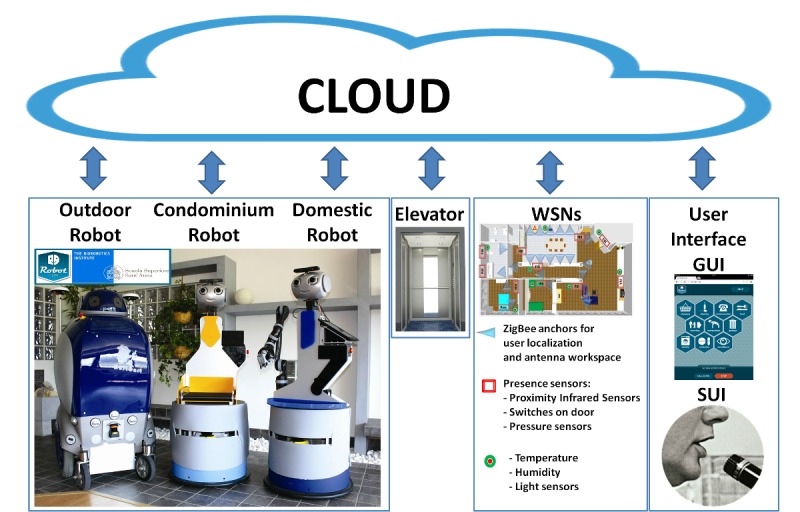
Robot-Era architecture. GUI: graphical user interface; SUI: speech user interface; WSN: wireless sensor network.

The Robot-Era system (Figure 1) implements six robotic services that involve three different environments: outdoor, condominium, and indoor. The agents involved in this system are the DOmestic RObot (DORO), COndominium RObot (CORO), Outdoor RObot (ORO), lift, wireless sensor networks (WSNs), graphical user interface (GUI), and speech interactions. All these agents are managed by a cloud platform based on elastic computing models in which resources are dynamically allocated from a shared resource pool in the cloud to support task offloading and information sharing in robotic applications [31].

#### DORO

This robot was developed on a SCITOS G5 platform (Metralabs, Germany) and safely navigates in a domestic environment. DORO can provide support to older individuals with its integrated robotic arm for object manipulation, tray for the transportation of objects, and handle for walking support. Furthermore, both visual and auditory feedback is provided to the user via multicolor LEDs mounted on the robot’s eyes, speakers, and GUI on a removable tablet.

#### CORO

The CORO robot works in the condominium environment and can navigate between floors using the elevator. It is equipped with a roller mechanism to exchange goods with ORO, and it provides feedback to users in the same manner as DORO.

#### ORO

This robot was designed on the DustCart platform and is an autonomous mobile robot for goods transportation in the urban environment by means of a container to carry the objects [32]. ORO has a head with multicolor LEDs in the eyes, a touchscreen on the left side, and speakers reproducing acoustic signals to provide information to the user.

#### Elevator

The elevator, already present in the environment, is embedded in the Robot-Era system through a Phidget input/output digital board used to control it remotely.

#### Wireless Sensor Networks

Two Zig-Bee WSNs are included in the Robot-Era system. The first network is designed for multiple user localization inside the domestic environment by observing the received signal strength. The second network was developed for home monitoring and passive localization of people. It consists of passive infraRed sensors, pressure sensors placed under a chair or bed, switches on doors or drawers, gas and water leak sensors, and sensors for temperature, humidity, and light.

#### The Graphical User Interface

**Figure 2:**
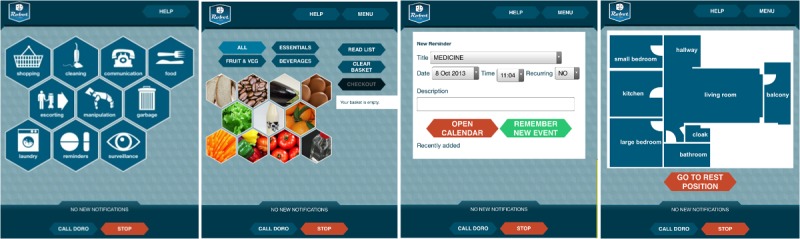
The Robot-Era graphical user interface.

A Web GUI (Figure 2), which runs on the robot’s tablet, is the GUI. A main menu index page allows the user to navigate between the different Robot-Era service pages that compose the GUI. The users can employ the GUI to call the robot, select a service, and perform the service [33].

#### Speech User Interface

Using the Bluetooth-connected wearable microphone, the user can ask for, and perform, a robotic service. Specifically, the robot can recognize certain keywords when a user is speaking, corresponding to the commands or the services that the robot can perform. The robot can perform speech synthesis through the speakers to interact with the user [34].

#### More Details

More details about the Robot-Era architecture are explained in [35].

### Robot-Era Services

The Robot-Era system can provide six advanced robotic services that were tested by real older users in Peccioli (Italy) to evaluate the usability and the acceptability of the system. The Robot-Era experiments were organized into two sessions. In the first session, the shopping, garbage collection, and communication services were tested. In the second session, the reminding, indoor walking support, and outdoor walking support services were examined.

#### Shopping Service

The older participant had to imagine they were sick and could not leave their home, but they needed several items to eat and drink. Bearing in mind this presupposition, the participants had to create and send a shopping list with five products using the GUI and wait for the shopping delivery. In this scenario, all three Robot-Era platforms were involved, working in three different environments.

#### Garbage Service

The older user wanted to dispose of garbage. The participant had to call the domestic robot to select the “garbage collection service.” Speech interaction or GUI could be used to accomplish this service.

#### Communication Service

This scenario consisted of two parts: a warning alert case and a phone call case. A gas leak inside the home was simulated and detected. The domestic robot went to the user to inform them about this dangerous situation. Immediately following the notification, an incoming call, by a possible caregiver, was visualized on the tablet and the user had to accept it. In the phone call case, the participant used the robot to call a family member via Skype. Users could use speech interaction and GUI to perform this service. Even if the communication service was composed of two parts, it was analyzed as a single service.

#### Reminding Service

The older user wanted to set a date on the Robot-Era agenda. The user called the domestic robot to perform the task, and then he or she moved to another room inside the home. The robot reached the user to remember the date. The speech and graphical interface interaction were necessary to perform this service.

#### Indoor Walking Support

The older user had to imagine that they had a temporary mobility problem, so they used the domestic robot as a walking support. The participant drove DORO using two buttons mounted on the handle.

#### Outdoor Walking Support

The user moved from point A to point B following a preset path and then returned. The individual used the joystick to drive the robot and then tried to open and close the robot bin, pushing the icon on the screen. In this scenario, only ORO worked in the outdoor environment.

### Participants

**Figure 3:**
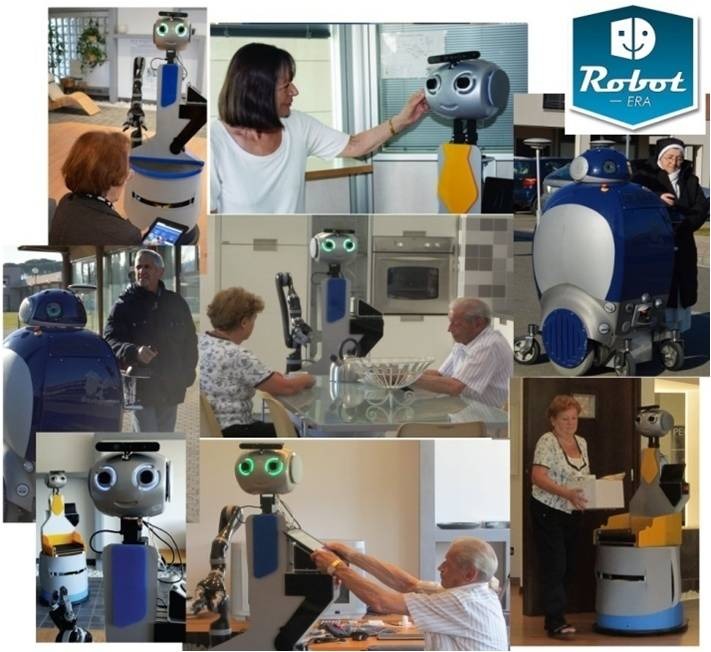
Participants involved in Robot-Era experimentation.

To recruit the needed older users, associations and groups working with senior people were contacted. Furthermore, the municipality of Peccioli sent an instructive brochure about the Robot-Era experimentation to all citizens older than 65 years of age. At the end of the recruitment phase, 45 older persons, aged between 65 and 86 years, were involved in the Robot-Era experimentation on a voluntary basis, and an informed consent was signed by each participant (Figure 3). To be enrolled in the study, the participants had to (1) be older than 65 years, (2) have a positive evaluation of mental status on (Short Portable Mental Status Questionnaire [SPSMQ]; cut-off errors ≤3) [36], and (3) have a minimum required autonomy in performing daily activities, evaluated with the Instrumental Activity of Daily Living Questionnaire (cut-off score >2) [37]. However, all participants made maximum two errors in answering to SPSMQ (cut-off errors ≤3), which means that they had normal mental functioning. Those who agreed to participate received a sociodemographic questionnaire. Given that the Robot-Era experimentation was organized in two sessions, older volunteers were grouped into two samples of 35 participants according to their availability. However, two participants did not complete the second experimental session, so they were eliminated from the study. Moreover, 23 participants participated both in the first experimentation session and in the second one 3 months later. The first sample was composed of 22 women and 13 men. Their mean age was 74.97 (SD 5.70) years and their achieved educational level was primary education for five participants, junior high school for five, high school for 20, and university for five. The second sample was composed of 22 women and 11 men. Their mean age was 73.45 (SD 6.27) years and their achieved educational level was primary education for 10 participants, junior high school for five, high school for 14, and university for four.

### Procedure

The experiments were conducted in Peccioli, Italy, and the overall system was used in three different environments: domestic, condominium, and outdoor. Each recruited participant was invited to the premises of the DomoCasa Lab, and the following experimental session was performed:

1. The Robot-Era project was introduced to the user by a researcher.
2. The user was free to gain confidence with the three robots, touching them and asking questions to clear up any confusion.
3. A questionnaire was given to the user to collect their first impressions about Robot-Era platforms.
4. A video tutorial in which a researcher assumed the role of an older user was shown to facilitate the understanding of the functioning and potentialities of the Robot-Era system.
5. The researcher announced the tasks of each Robot-Era service that the participant should fulfill via the robots. Subsequently, the user was asked whether they understood the tasks. If not, the action was repeated, and the tasks were explained again.
6. A written description of the tasks of each robotic service was given to the participant for them to refer to if needed as they tested the Robot-Era services.
7. The user performed each Robot-Era service.
8. The usability and acceptability of each robotics service were evaluated by the user through questionnaires.

During the experimental session, the older adult performed the test without assistance from the researcher to avoid any influence or bias. However, a researcher was present during the experiments for security issues, and the experimental session was video recorded.

### Evaluation Tools

One of the most important goals of robotics is to be able to give the robot the highest degree of acceptability. This concept plays a significant and delicate role in the industrial design, and in the context of robotics, this is even more pronounced. For this reason, a specific “appearance questionnaire” (Multimedia Appendix 2), based on a 5-point Likert scale, was developed to evaluate the impact of the robot’s appearance on the user. This questionnaire was designed to investigate:

1. Positive or negative feelings that could be evoked on seeing the Robot-Era robots for the first time (items A1-A2);
2. Robot-Era robots’ ability to arouse feelings of familiarity in the user thanks to their formal aspect, colors, and size (items A3-A8);
3. The perceived robustness of Robot-Era robots (items A9-A10);
4. Robot-Era robots’ ability to make their functions evident (items A11-A13); and
5. Robot-Era robots’ ability to establish a positive emotional relationship with the user (items A14-A15).
6. The appearance questionnaire was administered for each robot (DORO, CORO, and ORO).

For the services evaluation phase, an ad hoc questionnaire was developed, consisting of 14 items rated on a 5-point Likert scale (from totally disagree to totally agree; see Multimedia Appendix 3) and based on the following content:

1. Disposition about the Robot-Era services (items Q1-Q3);
2. Feelings of anxiety, enjoyment, and trust evoked using the robotics platforms (items Q4-Q8);
3. Perceived ease of use of the GUI during the performance of Robot-Era services (items Q9-Q11); and
4. Perceived ease of use of the speech user interface (SUI) during the performance of Robot-Era services (items Q12-Q14).

The choice of developing the original set of questions was motivated by the literature in the field of acceptability evaluation [38], which suggests the need for personalization of the tools to adjust the instrument to the specific technical features of the platform and the issues of interest for the project. Moreover, the development of an ad hoc tool represented a common practice for the psychosocial research. The psychometric proprieties of the appearance questionnaire and ad hoc questionnaire were assessed as detailed subsequently.

At the end of each tested service, the System Usability Scale (SUS) was administered to the volunteers to investigate the perceived usability of the Robot-Era services. The SUS is a survey instrument composed of 10 standardized items based on the 5-point Likert scale (from strongly disagree to strongly agree). It was developed according to the three usability criteria defined by the ISO 9241-11: (1) effectiveness: the ability of users to complete tasks using the system; (2) efficiency: the resources expended by users to achieve goals; and (3) satisfaction: the users’ subjective comfort using the system.

### Statistical Analysis

The first step was to estimate the reliability of the appearance questionnaire and the ad hoc questionnaire. Reliability was assessed as reliability over time and internal consistency reliability. Reliability over time of the ad hoc questionnaire was measured applying test-retest, because this tool was administered twice to the same 23 participants who were involved both in the first experimentation session and in the second one 3 months later. Regarding the appearance questionnaire, the test-retest was not applicable because this tool was administered one time. For this reason, the split-half method was applied dividing the tool into even and odd questions. The two halves of a measure were treated as alternate forms (same mean and standard deviation). Therefore, the correlation between the two halves was calculated as an estimate of the test-retest reliability. Finally, reliability estimate was stepped up to the full tool length using the Spearman-Brown prediction formula. The internal consistency reliability was assessed calculating the intraclass correlation coefficient (ICC) and Cronbach alpha.

For each questionnaire, the basic descriptive statistics were calculated: mean scores, standard deviation, and mode to obtain a first impression of the scores. Moreover, to obtain an overall score for each questionnaire, the sum of the item score contributions was rescaled from 0 to 100 because the 0 to 100 scale is more intuitive to understand. Furthermore, nonparametric tests were applied to compare different conditions and users. The choice of nonparametric statistics is necessary when the sample size is not large, and data are not normally distributed. The Mann-Whitney *U* test was used to compare men versus women and users younger than 75 years versus older than 75 years, whereas the Kruskal-Wallis test was used to compare different conditions in educational level and technology skill. Finally, the correlations among the appearance, ad hoc, and SUS questionnaires were investigated by calculating the Pearson correlation.

## Results

### Primary Findings

As shown in Multimedia Appendix 4 (Reliability of Questionnaires) about the appearance questionnaire administered for the DORO, CORO, and ORO robots, the split-half reliability, adjusted using the Spearman-Brown prophecy formula, was higher than .60 and *P*<.001; reliability over time higher than .40 is considered acceptable [39]. Regarding internal consistency reliability, the ICC was higher than .4; ICC values between .40 and 0.75 are good [40]. Moreover, Cronbach alpha value was higher than .60, which is considered acceptable for short instruments with a small number of items [41-43].

Considering the ad hoc questionnaire (Multimedia Appendix 4), test-retest reliability value (*r*=.68, *P*<.001) was acceptable [39] and internal consistency reliability was well estimated because ICC was higher than .40 [40] and Cronbach alpha was higher than .60 [41-43] for all Robot-Era services. In conclusion, the appearance and the ad hoc questionnaires could be considered reliable.

### Appearance Questionnaire Outcomes

**Figure 4:**
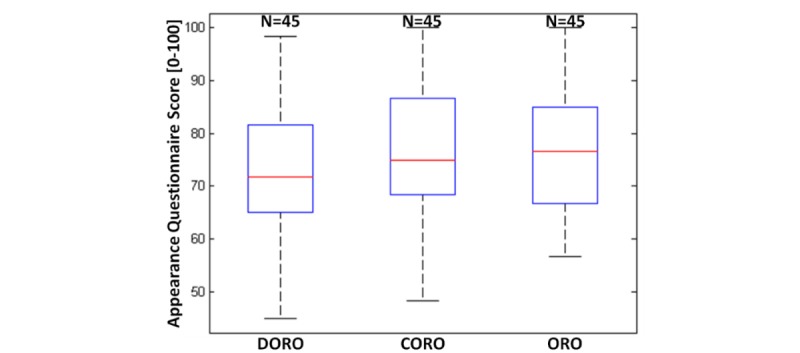
Boxplots of the overall scores, considered as the sum of the item score contributions, rescaled from 0 to 100, for the appearance questionnaires for the DOmestic RObot (DORO), COndominium RObot (CORO), and Outdoor RObot (ORO) systems. On each box, the central mark indicates the median, the bottom and top edges of the box the 25th and 75th percentiles, and the whiskers the most extreme data points not considered outliers.

Figure 4 reports the boxplot of the overall score: the mean values were 73.04 (SD 11.80) for DORO, 76.85 (SD 12.01) for CORO, and 75.93 (SD 11.67) for ORO.

In Table 1, descriptive statistics regarding the appearance questionnaire are reported. The results show that the items that were phrased negatively had a mean score lower than 3 and a mode value equal to 1 (except for item A8) related to DORO, with a mode value equal to 3. Conversely, the items that were phrased positively had a mean score greater than 3 with a mode value equal to 4 or 5. The only exceptions were items A3 and A10 with a mode value of 1 and 3, respectively.

Concerning the effect of gender, male participants gave ORO an overall score higher than female participants (*P*=.02). The appearance of ORO inspired more confidence in men than in women (item A2: *P=*.03). In addition, male participants had a higher propensity for touching and interacting with ORO than female participants (item A15: *P=*.048).

Regarding the impact of age, individuals younger than 75 years readily understood the functionalities of Robot-Era robots, more so than older people (item A11: *P*=.007 for DORO, *P*=.001 for CORO, and *P*=.046 for ORO).

Moreover, older users with a high educational level expressed willingness to interact with DORO (item A15: *P*=.007) and CORO (item A15: *P*=.047) more than volunteers with a low level of education.

Finally, older adults who were able to use a PC and the internet gave CORO and ORO a higher overall score than those who were not able to use such technologies (*P*=.03 for CORO and *P*=.01 for ORO).

### Ad Hoc Questionnaire Outcomes

**Figure 5:**
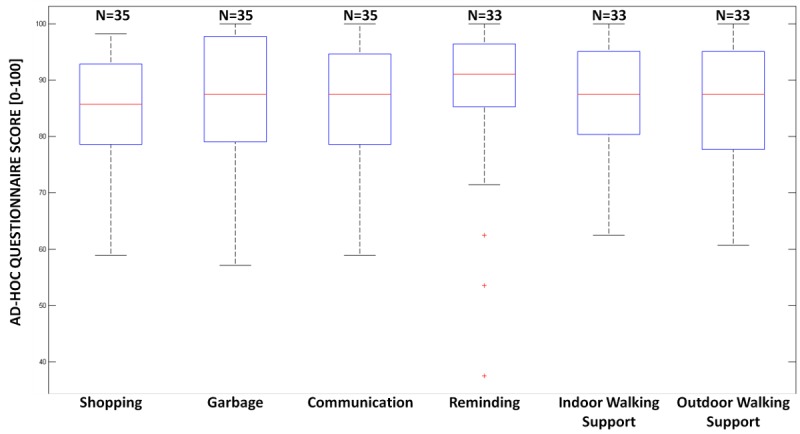
Boxplots of the overall scores, considered as the sum of the item score contributions, rescaled from 0 to 100, for the ad hoc questionnaire. On each box, the central mark indicates the median, the bottom and top edges of the box the 25th and 75th percentiles, and the whiskers the most extreme data points not considered outliers, and the outliers are plotted individually using the “+” symbol.

Regarding the results of the ad hoc questionnaire, the mean overall score was 84.59 (SD 10.32) for shopping, mean 87.30 (SD 10.84) for garbage, mean 86.73 (SD 9.11) for communication, mean 86.58 (SD 14.68) for reminding, mean 85.93 (SD 11.05) for indoor walking support, and mean 84.69 (SD 11.93) for outdoor walking support. Figure 5 shows the boxplot of the overall score.

Moreover, standard descriptive statistics presented a high rate of agreement, characterized by a high mean score for positively formulated items and a low mean score for negatively formulated items for all Robot-Era services (Table 2).

Concerning the effect of sociodemographic factors, participants with a high educational level gave Robot-Era services a higher score than those with a low level of education; specifically, for shopping (*P*=.04), garbage (*P*=.047), reminding (*P*=.04), indoor walking support (*P*=.006), and outdoor walking support (*P*=.03). Moreover, a significant difference was found between genders, because a higher score was given by male older adults for shopping (*P*=.02), indoor walking support (*P*=.02), and outdoor walking support (*P*=.03).

#### Shopping Service

Concerning the comparison between different conditions and users, men had more trust in the robot’s ability to perform the shopping service than women did (item Q7: *P=*.007). Regarding the age factor, the participants younger than 75 years would use the robot for shopping if necessary (item Q1: *P=*.04) and if it could reduce the family/caregiver’s work burden (item Q2: *P=*.04), more so than those older than 75 years. Moreover, participants with a high educational level thought that the proposed system could help the caregivers work less, more so than people with a low educational level (item Q2: *P*<.001). However, higher educated users had more trust in the robot’s ability to perform the shopping service (item Q7: *P=*.03) than less-educated users.

#### Garbage Collection Service

There was a significant difference in gender regarding the benefits that could lessen the family/caregiver’s work burden: men gave a higher score than did women (Item Q2: *P=*.02). Furthermore, more educated participants were more skeptical than less-educated ones about the help provided by the robotic system to caregivers (item Q2: *P=*.01). The more educated participants perceived the robot as less intrusive for privacy (item Q8: *P=*.03).

#### Communication Service

Men thought their independence would be improved using the communication service (item Q3: *P=*.03) more so than women. Furthermore, the robot was perceived as not intrusive (item Q8: *P=*.006) by men more so than by women. Furthermore, more males reported that it was easy to speak to the robot (item Q12: *P=*.047) than did females. The vocal commands to interact with the robot were understood (item Q13: *P=*.048) better by men than by women. Moreover, more participants younger than 75 years would use the Robot-Era system in case of need (item Q1: *P=*.04) than those older than 75 years. The younger group also felt the system could reduce the caregiver’s work burden more so than the older group did (item Q2: *P=*.04). Finally, individuals with a high educational level had a more positive attitude (item Q2: *P=*.001) and felt the robot was less intrusive (item Q8: *P=*.03) compared to the less-educated individuals.

#### Reminding Service

Participants’ independence could be increased by this service (item Q3: *P=*.047) to a larger extent for men than for women. Moreover, males recognized the icons to press on the tablet to perform the reminding service (item Q11: *P=*.03) better than the females did. Furthermore, more participants younger than 75 years reported that it was easier to use the speech commands (item Q12: *P=*.04; item Q13: *P=*.02) compared to those older than 75 years. Regarding educational level, more individuals with a high educational level thought this service could reduce the caregiver’s burden (item Q2: *P=*.02) and believed that the system was more reliable (Item Q7: *P=*.02) compared to participants with a low level of education.

**Table 1:** Descriptive statistics of items on the appearance questionnaire^a^ for the DOmestic RObot (DORO), COndominium RObot (CORO), and Outdoor RObot (ORO) systems (N=45).

System and questionnaire item^b^		Mean (SD)	Range	Mode
**DORO**				
	Item A1	1.18 (0.49)	1-3	1
	Item A2	4.33 (0.88)	1-5	5
	Item A3	2.98 (1.54)	1-5	1
	Item A4	4.09 (0.95)	1-5	5
	Item A5	4.27 (0.86)	2-5	5
	Item A6	2.02 (1.27)	1-5	1
	Item A7	4.22 (0.79)	2-5	5
	Item A8	3.07 (1.37)	1-5	3
	Item A9	4.18 (0.81)	1-5	4
	Item A10	3.73 (1.03)	1-5	3
	Item A11	2.62 (1.37)	1-5	1
	Item A12	3.82 (1.11)	1-5	5
	Item A13	2.22 (1.68)	1-5	1
	Item A14	1.49 (1.08)	1-5	1
	Item A15	3.82 (1.25)	1-5	5
**CORO**				
	Item A1	1.11 (0.32)	1-2	1
	Item A2	4.31 (0.95)	1-5	5
	Item A3	2.76 (1.43)	1-5	1
	Item A4	4.24 (0.80)	2-5	5
	Item A5	4.64 (0.48)	4-5	5
	Item A6	1.67 (1.15)	1-5	1
	Item A7	4.33 (0.88)	1-5	5
	Item A8	2.11 (1.34)	1-5	1
	Item A9	4.22 (0.79)	1-5	4
	Item A10	3.84 (0.98)	1-5	3
	Item A11	2.69 (1.33)	1-5	1
	Item A12	4.11 (0.83)	2-5	4
	Item A13	2.16 (1.64)	1-5	1
	Item A14	1.49 (1.08)	1-5	1
	Item A15	3.87 (1.18)	1-5	5
**ORO**				
	Item A1	1.24 (0.61)	1-3	1
	Item A2	4.24 (0.96)	1-5	5
	Item A3	2.56 (1.39)	1-5	1
	Item A4	3.93 (0.94)	2-5	5
	Item A5	4.42 (0.87)	1-5	5
	Item A6	1.89 (1.27)	1-5	1
	Item A7	4.53 (0.66)	3-5	5
	Item A8	1.73 (1.34)	1-5	1
	Item A9	4.40 (0.81)	1-5	4
	Item A10	3.84 (0.98)	1-5	3
	Item A11	2.78 (1.43)	1-5	1
	Item A12	4.16 (0.80)	3-5	4
	Item A13	2.20 (1.69)	1-5	1
	Item A14	1.53 (1.10)	1-5	1
	Item A15	3.84 (1.15)	1-5	5

^a^See Multimedia Appendix 2 (Appearance questionnaire).

^b^A1: the robot looks dangerous; A2: the appearance inspires confidence in me; A3: the appearance is familiar; A4: the appearance is aesthetically pleasing; A5: the colors are appropriate; A6: the appearance is out of proportion and nonsymmetric; A7: the appearance is in good agreement; A8: the robot is too big and bulky; A9: the complete robot and its various parts seem robust; A10: the materials are appropriate; A11: the appearance is unable to communicate its functions; A12: the position of the touchscreen is appropriate; A13: the presence of colored lights in the eyes of the robot is useless; A14: the presence of a head on the robot restricts or inhibits the interaction with the robot; A15: the appearance invites me to touch and interact with it.

#### Indoor Walking Support Service

Men had a more positive attitude toward this robotic service (item Q1: *P=*.04; item Q3: *P=*.004) than women did. Furthermore, more educated participants had more trust in the ability of the Robot-Era system (item Q7: *P=*.04) than those with a lower level of education.

#### Outdoor Walking Support Service

More men felt that their independence could be improved by this service (item Q3: *P=*.03) than women did.

### Comparing Questionnaires

Investigating the correlation among the questionnaires, there were significant results between the appearance questionnaire related to DORO and the ad hoc questionnaire for shopping (*r*=.35, *P*=.04), communication (*r*=.41, *P*=.02), reminding (*r*=.35, *P*=.04), and indoor walking support (*r*=.35, *P*=.04) services, whereas there was not a significant correlation between the appearance questionnaire and the SUS. Finally, the ad hoc questionnaire and SUS were correlated for all Robot-Era services: shopping (*r*=.65, *P*<.001), garbage (*r*=.43, *P*=.01), communication (*r*=.41, *P*=.001), reminding (*r*=.71, *P*<.001), indoor walking support (*r*=.37, *P*=.04), and outdoor walking support (*r*=.39, *P*=.03)

**Table 2:** Descriptive statistics for the ad hoc questionnaire^a^ for services.

Service		Mean (SD)	Range	Mode
**Shopping service**				
	Item Q1	4.66 (0.94)	1-5	5
	Item Q2	4.49 (1.07)	1-5	5
	Item Q3	3.69 (1.53)	1-5	5
	Item Q4	1.20 (0.68)	1-4	1
	Item Q5	1.14 (0.69)	1-5	1
	Item Q6	4.46 (1.04)	1-5	5
	Item Q7	4.54 (0.66)	3-5	5
	Item Q8	1.09 (0.37)	1-3	1
	Item Q9	3.49 (1.34)	1-5	3
	Item Q10	3.86 (1.35)	1-5	5
**Garbage service**				
	Item Q1	4.69 (0.90)	1-5	5
	Item Q2	4.54 (0.98)	1-5	5
	Item Q3	4.14 (1.33)	1-5	5
	Item Q4	1.14 (0.69)	1-5	1
	Item Q5	1.11 (0.68)	1-5	1
	Item Q6	4.46 (1.07)	1-5	5
	Item Q7	4.74 (0.56)	3-5	5
	Item Q8	1.20 (0.76)	1-5	1
	Item Q9	3.94 (1.19)	1-5	5
	Item Q10	4.17 (1.07)	2-5	5
**Communication service**				
	Item Q1	4.63 (0.91)	1-5	5
	Item Q2	4.34 (0.97)	1-5	5
	Item Q3	3.91 (1.34)	1-5	5
	Item Q4	1.17 (0.62)	1-4	1
	Item Q5	1.14 (0.49)	1-3	1
	Item Q6	4.57 (1.01)	1-5	5
	Item Q7	4.43 (0.88)	1-5	5
	Item Q8	1.26 (0.95)	1-5	1
	Item Q9	4.09 (1.22)	1-5	5
	Item Q10	4.23 (1.26)	1-5	5
**Reminding service**				
	Item Q1	4.55 (1.09)	1-5	5
	Item Q2	4.48 (1.23)	1-5	5
	Item Q3	4.27 (1.21)	1-5	5
	Item Q4	1.12 (0.55)	1-4	1
	Item Q5	1.24 (0.66)	1-4	1
	Item Q6	4.30 (1.24)	1-5	5
	Item Q7	4.55 (1.03)	1-5	5
	Item Q8	1.33 (1.08)	1-5	1
	Item Q9	3.76 (1.39)	1-5	5
	Item Q10	4.61 (0.79)	2-5	5
**Indoor walking support service**				
	Item Q1	4.45 (1.23)	1-5	5
	Item Q2	4.55 (1.12)	1-5	5
	Item Q3	3.58 (1.58)	1-5	5
	Item Q4	1.03 (0.17)	1-2	1
	Item Q5	1.00 (0.00)	1-1	1
	Item Q6	4.61 (0.79)	3-5	5
	Item Q7	4.61 (0.83)	1-5	5
	Item Q8	1.42 (1.06)	1-5	1
	Item Q9	3.76 (1.39)	1-5	5
	Item Q10	4.61 (0.79)	2-5	5
**Outdoor walking support service**				
	Item Q1	4.48 (1.23)	1-5	5
	Item Q2	4.33 (1.24)	1-5	5
	Item Q3	4.03 (1.31)	1-5	5
	Item Q4	1.27 (0.91)	1-5	1
	Item Q5	1.00 (0.00)	1-1	1
	Item Q6	4.48 (0.87)	1-5	5
	Item Q7	4.36 (1.06)	1-5	5
	Item Q8	1.76 (1.35)	1-5	1
	Item Q9	3.76 (1.39)	1-5	5
	Item Q10	4.61 (0.79)	2-5	5

^a^See Multimedia Appendix 3 (Ad hoc questionnaire).

## Discussion

### Principal Results Regarding Robot’s Appearance

New technologies are increasingly impacting the entire society, but older adults often have difficulty accepting them. This reluctance could be due to the fear of trying something new, not perceiving the need for the technology, and the lack of training to use new technologies [44-46]. Moreover, many older individuals have never experienced such technologies, or at least they benefit from them to a lesser extent than younger people [47]. In this study, participants were free to become familiar with the Robot-Era robots before starting the experiment session to feel more confident in testing them. A video tutorial was shown to illustrate all Robot-Era services and older volunteers could touch the robots and ask questions about their functionalities to become confident with them. In fact, adequate training can increase the level of acceptance [48].

Participants had quite a positive impression of Robot-Era robots, as shown by the median score of 71.67 for DORO’s appearance, 75.00 for CORO, and 76.67 for ORO. Furthermore, there was an upward trend in median score related to the workplace environment of the robot, as confirmed by the increase of the minimum value of the overall score (see Figure 4). Looking at these data, older adults tend to express a more positive opinion about CORO and ORO, which usually do not live in the domestic environment with humans but work in condominium and urban areas, respectively. A conscious and total acceptance of a robot in a domestic environment could reflect the successful diffusion of robots within society, starting from the outdoor environment and progressing to their incorporation in the private house. This hypothesis finds a confirmation in the fact that older volunteers, able to use a PC and the internet, gave a higher score to CORO and ORO than those individuals who were not able to use these technologies. The older adults with technology experience were aware that these technologies can connect the outside world and their own homes, such as CORO and ORO are able to do. Moreover, ORO received a higher score by men than women because more male participants reported that the outdoor robot had a masculine aspect than female participants did.

The appearance of a robot is a factor that may impact human-robot interaction and acceptance by older adults, even if older people did not express any preferences regarding the robot’s appearance [49]. Furthermore, a human-like robot can confuse older individuals, so in the Robot-Era project, the choice was a mixed appearance between the anthropomorphic and machine features since all robots are equipped with a motorized head. The head is characterized by blinking colored eyes, a stylized mouth, and two small, soft disks on the side that resemble ears. Watching the Robot-Era robots for the first time, all participants said something like, “They have a nice face,” “They are smiling,” or “They are welcoming.” These sentences confirm that the older volunteers were positively impressed and, in effect, that facial features of the robots—especially nose, eyelids, and mouth—can positively influence acceptance [50]. In fact, 40 of 45 older adults thought that the presence of a head on the robot promotes interaction with it (Table 1, item A14).

Furthermore, the Robot-Era robots are developed with a height of 1.50 m, which is shorter than an average human adult’s height, for the user to perceive having control over the robot without feeling dominated by it. Thanks to this choice and the presence of a head, DORO, CORO, and ORO do not evoke negative reactions in older users because they are judged not dangerous and they inspire confidence, as confirmed respectively by the low average score of item A1 (A1: the robot looks dangerous) and the high score of item A2 (A2: the appearance of the robot inspires confidence in me); see Table 1. Moreover, the acceptance of new technologies increases if they are familiar with something known by end users. For this reason, the shape of Robot-Era robots is designed to remind users of a domestic worker for DORO, a janitor for CORO, and a delivery man for ORO. Unfortunately, this goal was not reached as shown by the low score of item A3 (A3: the appearance of the robot is familiar to me); see Table 1. The justification of this low familiarity may not necessarily imply disliking or rejection of the robots, but it could mean that people do not ever like innovation or creativity.

Moreover, Robot-Era robots have to share and coexist with humans, so they have to integrate themselves in real environments from an esthetic and functional point of view. Investigating this issue, the survey outcomes show that DORO’s appearance was pleasing for 34 of 45 older adults, CORO’s for 37 users, and ORO’s for 34 (Table 1, item A4). Additionally, the colors of the three robots are appropriate as confirmed by the high average score of item A5 (Table 1). Considering that, it is reasonable to think that Robot-Era robots could fit well within a domestic, condominium, and outdoor environment as demonstrated by the positive results of item A7 (Table 1). Furthermore, the size of a robot is an important perspective because it has to give the impression to work efficiently without damaging the environment. According to older individuals’ feedback, CORO and ORO are not perceived as too big or bulky compared, respectively, to a condominium and outdoor environment (Table 1, item A8). However, the participants assumed a neutral position regarding DORO’s size (Table 1, item A8) because most of them lived in a small house, but they were open to changing their minds after watching it move in a domestic environment.

The appearance of a robot should be perceived as robust to people who should have trust in it. Investigating this issue, Robot-Era robots and their various components seem sufficiently robust according to the positive feedback from older individuals for item A9 and item A10 (Table 1). However, all participants reported that they were not competent to judge this point, and they gave a high score, saying they trusted the developers.

Furthermore, a robot should be clearly understandable and easy to use to be accepted by end users. According to the survey outcomes, all Robot-Era robots can successfully communicate their functions as confirmed by item A11 (Table 1) and colored lights in the eyes of the robots were judged useful to communicate (Table 1, item A13).

Individuals younger than 75 years readily understood the functionalities of Robot-Era robots, more so than older individuals, likely because the younger volunteers lead a more active life, so they are more familiar with new technologies, such as tablets and smartphones, which are achieving market and society penetration. Furthermore, the high score of item A12 confirms that the position of the tablet is perfect for its use for all robots.

Finally, according to the results for item A15, the appearance of the Robot-Era robots invites the user to touch and interact with them. Moreover, older users with a high educational level expressed a greater willingness to interact with DORO and CORO, possibly because they are open, due to their educational background, to perceiving the robot as a social entity.

### Principal Results of the Ad Hoc Questionnaire

Looking at Figure 5, Robot-Era services were acceptable by older adults because the majority of the sample gave an overall score higher than 75 points, and the high degree of acceptance is also confirmed by the positive results shown in Table 2. The acceptance of robots by older people is related to their attitude toward robots because attitude is an important factor to understand the intention to use any technology [51]. In this study, the outcomes of the survey show a positive attitude toward Robot-Era services because the mean scores of item Q1 and item Q2 were higher than 4 and the mode was equal to 5 for all services. As matter of fact, all participants reported that they would share their life with a robot if the time came when they would not be able to perform their daily tasks. Moreover, many volunteers said they would prefer to be assisted by a robot to avoid burdening their sons and daughters with their care. Furthermore, Robot-Era services have the potential to improve the independence of older people, as confirmed by the high mean score and mode equal to 5 for item Q3. Many older adults reported that the Robot-Era system could prevent them from having to do boring tasks such as taking out the trash. Moreover, most of the participants said they would feel safer in their own homes using the Robot-Era services because DORO is able to communicate alert messages such as “There is a gas leak” or “The door is open” and because the robotics system can call a caregiver automatically in the event of dangerous situations. Furthermore, the capabilities of DORO to locate the user in the house and to remind them to take their medicine were much appreciated by older adults who would no longer need to worry about forgetting to take their medications thanks to this robotic service. According to the feedback from older users, the indoor walking support service is useful to move safely in the home thanks to the robot’s handle. However, the mean score of item Q3 was not too high because the participants did not have mobility impairments. Nevertheless, they would use DORO to transport objects or laundry from one room to another, taking advantage of the robot’s capabilities to navigate autonomously, because older users said they would feel safer if the robot would do that task for them, so they would avoid the risk of falls during this task. The same arguments are valid for the outdoor walking support service. In addition, the older participants would like the social capability of the outdoor robot to be improved. Furthermore, according to participants, the shopping need was not perceived as a burdensome task, but as a socialization means; however, they said that this service is useful in the case of temporary mobility impairments or bad weather.

Anxiety toward robots is an important issue to be faced, and often older adults have negative feelings about the idea of having a robot assistant, particularly in a home environment [52]. Conversely, the Robot-Era system did not evoke anxious or negative emotional reactions in older participants during the experimentation because almost no one was embarrassed or nervous when interacting with the robots, as confirmed by a low score of item Q4 and item Q5. Furthermore, many participants expressed that, before starting the experiments, they were worried about appearing inadequate should they not be able to complete the test. However, they said they felt relaxed and comfortable thanks to the explanations provided by the researchers in the starting phase. In effect, the participants enjoyed using the Robot-Era system, as confirmed by the high agreement with item Q6. Only two users did not get pleasure in testing the Robot-Era system because they claimed to see the robotics system as an appliance that is used for its usefulness and not for pleasure. Furthermore, the trust in the ability of the Robot-Era system to perform with integrity and reliability is a factor that affects the acceptance, and the participants expressed a high degree of trust in the Robot-Era system (item Q7). The older adults justified their answers, saying that all provided robotic services were successful during the experimentations. Moreover, the development of robotic systems working in daily living environments raises ethical issues such as privacy problems. However, according to the older volunteers, the Robot-Era system was not too intrusive for their privacy, as confirmed by the low score obtained for item Q8. Some participants said that their privacy would not be a concern since they can freely choose whether or not to use the proposed robotic services. Other older adults said that the Robot-Era system was not more intrusive than other technologies, whereas some male participants joked that a robot is less intrusive than their wives. Regarding the items related to the perceived ease of use of GUI, the feedback of participants was quite positive, and it should be considered that most of them did not have familiarity with the tablet and they had some starting difficulty because it was the first time they used it. In particular, the tablet was found easy to use (item Q9), the messages on it were read (item Q10), and the icons to perform the services were identified (item Q11). Therefore, at the end of the experiments, the older adults gave some suggestions to improve the GUI such as adding the captions to the icons. However, everybody reported a willingness to learn to use the tablet because it has widespread use in society. Finally, the speech interaction was well evaluated by older users because they spoke to the robot easily (item Q12), they understood the vocal commands to interact with the robot (item Q13), and they heard without any major difficulties what the robot said (item Q14). Moreover, the participants reported that they enjoyed speaking to the robot because it was seen as the more natural means to interact with it. Although the robot communicated in quite a sophisticated manner, it did not understand if a synonym of the keywords was used. For this reason, the participants suggested increasing the vocabulary of the robot, so that the user could speak in a natural way without having to remember the keywords to use. Moreover, the older adults suggested that the robot should give more feedback about its status, such as describing what it is doing, and the robot should communicate to the user if it understood a command.

Concerning the effect of sociodemographic factors, it seems that men have a more positive attitude toward Robot-Era services and, in effect, men are less skeptical in using assistive robotic technologies than women [53] and they have a more positive attitude than women toward the possibility of using a robot in the future [54]. As shown in the previous section, gender could have an impact on the acceptance of the technology. Examples of this in the study are that men would use the indoor walking support, in case of need, more than women (item Q1), and regarding the garbage collection service, male participants thought that the Robot-Era system could reduce the caregiver’s work burden (item Q2). Furthermore, communication, reminding, indoor walking support, and outdoor walking support could improve men’s independence more than women’s (item Q3). The trust in the robot’s ability to perform the shopping service (item Q7) was higher in males than in females, who also thought a robot would be too intrusive for their privacy (item Q7, communication). In general, men seem more willing to accept robotic technologies in their daily lives than women [55]. Furthermore, men perceived the interaction modalities (item Q11: reminding, indoor walking support, and outdoor walking; item Q12 and item Q13: communication) as easier than women did because males tend to be more task-oriented and motivated to achieve specific goals [56].

Regarding the effect of age on attitudes toward technology, acceptance decreases with increasing age and young older users are more likely to use technology [57]. However, if technology meets the older individuals’ needs, the effect of age on acceptance becomes less important [58]. In this study, the results show that older users positively evaluated Robot-Era services regardless of age, except for the shopping and communication services, in which the participants younger than 75 years, more than those older than 75 years, would use the Robot-Era system in case of need (item Q1) and if it could reduce the caregiver’s work burden (item Q2). Furthermore, the speech commands to perform the reminding service were evaluated as easier to use by young older users than older ones (item Q12 and item Q13). These results can be explained on the basis of cultural background because the sense of family ties is very strong for people older than 75 years, who think they should be assisted by their sons and daughters. Moreover, younger people placed more trust in technology because they were more familiar with it, whereas the older individuals thought that the new technologies were far too complicated [53].

Concerning the factor of education level, it was found that people with a high education level expressed a positive attitude toward a robot [53]. However, in this study, the participants with a higher education level tended to have a less positive attitude toward the shopping (item Q1, item Q2) and garbage collection (item Q2) services than those who had a low educational level. This could be explained by the fact that the participants with a higher education level tended to live in towns where they had more access to services such as home grocery delivery and curbside collection. Alternatively, participants who lived in rural areas, where these services were less widespread, needed a family member’s help for transportation of goods and for this reason they would like to use robotic service to relieve the caregiver of these duties. However, in keeping with their familiarity with advanced technologies, older users with a high educational level reported more positive judgments about communication (item Q2) and reminding (item Q2) services. Furthermore, individuals with a higher education level had more trust in the robot’s ability to perform shopping (item Q7) and reminding (item Q7, *P*=.02), and felt that the robot was not intrusive for their privacy.

However, even if some correlations between sociodemographic factors and the ad hoc questionnaire items were highlighted, the Robot-Era system could be considered acceptable by a large segment of the older population.

Finally, the significant correlation between the appearance questionnaire related to DORO and the ad hoc questionnaire for shopping, communication, reminding, and indoor walking support services suggests that the acceptance by older users could be influenced and increased by the positive impression aroused by the esthetics of a robot. However, it should be considered that DORO was the robotic platform the older adults interacted with for more time during the experimentation.

### Strengths and Limitations

The strength of this study is that it reflects the real users’ perceptions of acceptability of services provided by a robotic system. The rationale is that 35 older adults tested six robotic services in realistic environments; moreover, the individuals worked with three robots in a domestic, condominium, and outdoor environment to guarantee the continuity of the robotic services from private houses to public areas and vice versa.

The study had some limitations. First, the appearance and the ad hoc questionnaires were developed specifically for the Robot-Era experiments, but they were not pilot tested nor validated before the trial sessions were started. However, the internal consistency was verified by applying the Cronbach alpha test and all questionnaires had an alpha value higher than .60.

Second, the Robot-Era experimentation was organized in two sessions, testing three services at a time. In this respect, the two samples were not composed of the same participants because some of the participants were not available to participate in both experimental sessions. Furthermore, the sample was not sex-balanced, but this is because, at the age of 65 years, women in Europe have a life expectancy higher than men.

Third, participants spent 3 hours testing the Robot-Era system during which time they alternated the testing of each robotic service and the evaluation phase. This adopted experimentation format brought a lack of continuity that could have given an incomplete overview of the robotic services and prevented its potential from being fully explored. In each case, this experimentation was positively used to gather feedback to improve the Robot-Era system. In the future, participants should interact with the robots for longer and in a more realistic setting, postponing the evaluation phase to the end of the trials.

Fourth, during the trial, some technical problems occurred, and this could have biased the user’s perception of the robotic system. For further trials, the dependability of the Robot-Era system should be improved so that older adults can evaluate a reliable robotic system.

Finally, the recruitment was limited to older persons who lived in Peccioli Municipality, a small village in the Italian countryside, so the catchment area covered a small number of older citizens. Furthermore, only participants without cognitive and physical impairments were recruited because the Robot-Era system was conceived for frail older persons living alone at home without a formal caregiver’s support. For this reason, the randomization of the sample was not feasible.

### Conclusion

This paper presents the results of a realistic experimentation of a robotic system for supporting independent living of older people. The approach overcomes some of the limitations of previous similar experiments. Six robotic services were tested by a total of 35 older users, who directly interacted with three autonomous robots, which cooperated between them in smart environments to accomplish everyday life tasks.

Looking at the proposed robotics system, interesting outcomes were found. In general, the Robot-Era robots’ esthetic and functionalities had a positive impact on the older adults, as shown by the high scores they gave to DORO, CORO, and ORO. Moreover, the results suggest that the positive perception of the robots’ esthetics could play a role in increasing the acceptance of robotic services by older persons.

Finally, according to all aspects discussed in this work and based on the feedback given by the end users, the Robot-Era system has the potential to be developed as a socially acceptable and believable provider of robotic services to promote the ability for older individuals to remain in their homes. Future works will foresee experimentations with the involvement of users with mild functional impairments.

## Appendix

Multimedia Appendix 1 Overview of related works.

Multimedia Appendix 2 Appearance questionnaire.

Multimedia Appendix 3 Ad-hoc questionnaire.

Multimedia Appendix 4 Reliability of questionnaires.

Multimedia Appendix 5 Video of scenarios.

## Abbreviations

CORO: COndominium RObot
DORO: DOmestic RObot
GUI: graphical user interface
ICC: intraclass correlation coefficient
ORO: Outdoor RObot
SPSMQ: Short Portable Mental Status Questionnaire
SUI: speech user interface
SUS: System Usability Scale
WSN: wireless sensor networks
