# Supplementary material for: Robotic Services Acceptance in Smart Environments With Older Adults: User Satisfaction and Acceptability Study
Source: J Med Internet Res. 2018 Sep 21;20(9):e264. doi: 10.2196/jmir.9460 (PMC6231879; doi:10.2196/jmir.9460)
Supplement: Multimedia Appendix 1 [file jmir_v20i9e264_app1.pdf]

Overview of Related Works

Study	Participants	User-Robot Interaction	Evaluation tools	Robot	Methodology
[18]	22	No	Interviews and questionnaires	No	Pictures of robots
[19]	20	No	Focus group discussions and semi-structured interviews	No	Pictures of robots<br>Video clips
[20]	18	No	Semi-structured focus group and questionnaires	RobuLAB 10	Live robot demonstration
[21]	14	No	Focus group discussion and questionnaires	Tele-presence Kubi and Beam robots	Video clips
[22]	49	Yes	Interviews and questionnaires	Hobbit PT1	Remote controlled robot
[24]	16	Yes	Questionnaires	DARCI robot	M1 autonomous mobile manipulator
[25]	16	Yes	Questionnaires	ASTROMOBILE	Autonomous robot
[26]	30	Yes	Interviews	Tele-presence robot	Tele-operated by user
[27]	25	Yes	Questionnaires	iRobi	Stationary robot
[29]	2	Yes	Qualitative tools and Questionnaires	Giraff	Tele-operated
