# Supplementary material for: Robotic Services Acceptance in Smart Environments With Older Adults: User Satisfaction and Acceptability Study
Source: J Med Internet Res. 2018 Sep 21;20(9):e264. doi: 10.2196/jmir.9460 (PMC6231879; doi:10.2196/jmir.9460)
Supplement: Multimedia Appendix 2 [file jmir_v20i9e264_app2.pdf]

Appearance Questionnaire

Please, Rate your opinion using a scale from 1 = strongly disagree to 5 = strongly agree

		DORO	CORO	DUSTCART
		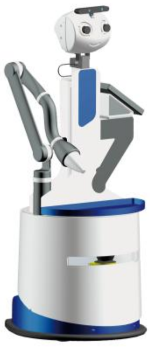	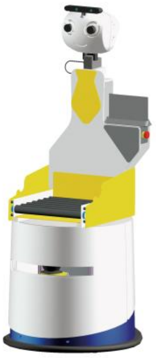	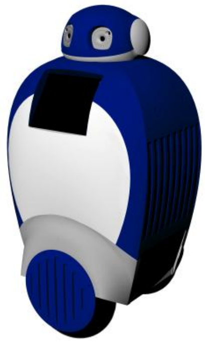
**Item A1**	The robot looks dangerous	1 2 3 4 5	1 2 3 4 5	1 2 3 4 5
**Item A2**	The appearance of the robot inspires confidence in me	1 2 3 4 5	1 2 3 4 5	1 2 3 4 5
**Item A3**	The appearance of the robot is familiar to me because it reminds me of	...a domestic worker 1 2 3 4 5	... a janitor 1 2 3 4 5	... a delivery man 1 2 3 4 5
**Item A4**	The appearance of the robot is aesthetically pleasing	1 2 3 4 5	1 2 3 4 5	1 2 3 4 5
**Item A5**	The colors of the robot are appropriate	1 2 3 4 5	1 2 3 4 5	1 2 3 4 5
**Item A6**	The appearance of the robot is out of proportion and non-symmetric	1 2 3 4 5	1 2 3 4 5	1 2 3 4 5
**Item A7**	The appearance of the robot is in good agreement with	... a domestic environment 1 2 3 4 5	... a condominium environment 1 2 3 4 5	... an outdoor environment 1 2 3 4 5
**Item A8**	The robot is too big and bulky compared to the environment	1 2 3 4 5	1 2 3 4 5	1 2 3 4 5
**Item A9**	The complete robot and its various parts seem robust	1 2 3 4 5	1 2 3 4 5	1 2 3 4 5
**Item A10**	The materials that make up the robot are appropriate	1 2 3 4 5	1 2 3 4 5	1 2 3 4 5
**Item A11**	The appearance of the robot is unable to communicate its functions	1 2 3 4 5	1 2 3 4 5	1 2 3 4 5
**Item A12**	The position of the touch-screen is perfect for its use	1 2 3 4 5	1 2 3 4 5	1 2 3 4 5
**Item A13**	The presence of coloured lights in the eyes of the robot is useless and tells me nothing	1 2 3 4 5	1 2 3 4 5	1 2 3 4 5
**Item A14**	The presence of a head on the robot restricts or inhibits the interaction with the robot	1 2 3 4 5	1 2 3 4 5	1 2 3 4 5
**Item A15**	The appearance of the robot invites me to touch and interact with it	1 2 3 4 5	1 2 3 4 5	1 2 3 4 5
