# Supplementary material for: Robotic Services Acceptance in Smart Environments With Older Adults: User Satisfaction and Acceptability Study
Source: J Med Internet Res. 2018 Sep 21;20(9):e264. doi: 10.2196/jmir.9460 (PMC6231879; doi:10.2196/jmir.9460)
Supplement: Multimedia Appendix 3 [file jmir_v20i9e264_app3.pdf]

Ad-Hoc Questionnaire

Please, Rate your opinion using a scale from 1 = strongly disagree to 5 = strongly agree
1=Strongly disagree, 2=Disagree, 3=Neither agree nor disagree, 4=Agree, 5=Strongly agree

**Item Q1**	I would use the robot for doing [a]...., in case of need (i.e. if I was sick)	1	2	3	4	5
**Item Q2**	I would be willing to use the [a].... service if it could help the family/caregiver's work.	1	2	3	4	5
**Item Q3**	I think my independence would be improved by the use of the robot for [a].....	1	2	3	4	5
**Item Q4**	I was too embarrassed in using the robot, around the community or the family.	1	2	3	4	5
**Item Q5**	I was nervous doing the [a].... service with the robot.	1	2	3	4	5
**Item Q6**	I enjoyed using the robot for doing [a]..... service	1	2	3	4	5
**Item Q7**	I would trust in the robot's ability to perform [a].... service.	1	2	3	4	5
**Item Q8**	If the robot shopped for me, it would be too intrusive for my privacy.	1	2	3	4	5
**Item Q9**	I found the tablet easy to use to perform the [a].... service.	1	2	3	4	5
**Item Q10**	I could clearly read the messages on the tablet.	1	2	3	4	5
**Item Q11**	I understood what buttons I needed to press to perform the [a].... service.	1	2	3	4	5
**Item Q12**	I found it easy to speak to the robot to perform the [a].... service.	1	2	3	4	5
**Item Q13**	I understood what I could say to the robot to perform the [a].... service.	1	2	3	4	5
**Item Q14**	I could clearly hear what the robot said to me.	1	2	3	4	5

[a] shopping/ garbage/ communication/ reminding/ indoor walking support/ outdoor walking support
