# Supplementary material for: Robotic Services Acceptance in Smart Environments With Older Adults: User Satisfaction and Acceptability Study
Source: J Med Internet Res. 2018 Sep 21;20(9):e264. doi: 10.2196/jmir.9460 (PMC6231879; doi:10.2196/jmir.9460)
Supplement: Multimedia Appendix 4 [file jmir_v20i9e264_app4.pdf]

Questionnaires Reliability

	r[a]	SB Correction[b]	*P* value	ICC[c]	α[d]
**Appearance Questionnaire**					
DORO	.48	.66	<.001	.46	.65
CORO	.56	.72	<.001	.56	.72
ORO	.62	.76	<.001	.54	.67
**Ad-Hoc Questionnaire**					
Shopping Service				.67	.66
Garbage Service				.70	.73
Communication Service				.67	.65
Reminding Service				.64	.85
Indoor walking support Service				.66	.69
Outdoor walking support Service				.60	.71

[a]Split-half reliability
[b]Split-half reliability adjusted using the Spearman–Brown prophecy formula
[c]intraclass correlation coefficient
[d]Cronbach's Alpha[e]
